# Absence of synergy for monosynaptic Group I inputs between abdominal and internal intercostal motoneurons

**DOI:** 10.1152/jn.00245.2014

**Published:** 2014-06-11

**Authors:** T. W. Ford, C. F. Meehan, P. A. Kirkwood

**Affiliations:** ^1^Sobell Department for Motor Neuroscience and Movement Disorders, UCL Institute of Neurology, London, United Kingdom;; ^2^University of Nottingham School of Health Sciences, Queen's Medical Centre, Nottingham, United Kingdom; and; ^3^Department of Neuroscience and Pharmacology, Panum Institute, Copenhagen N, Denmark

**Keywords:** Group I inputs, thoracic spinal cord, motoneurons, muscle synergies, respiration, posture, abdominal muscles, intercostal muscles

## Abstract

Internal intercostal and abdominal motoneurons are strongly coactivated during expiration. We investigated whether that synergy was paralleled by synergistic Group I reflex excitation. Intracellular recordings were made from motoneurons of the internal intercostal nerve of T_8_ in anesthetized cats, and the specificity of the monosynaptic connections from afferents in each of the two main branches of this nerve was investigated. Motoneurons were shown by antidromic excitation to innervate three muscle groups: external abdominal oblique [EO; innervated by the lateral branch (Lat)], the region of the internal intercostal muscle proximal to the branch point (IIm), and muscles innervated from the distal remainder (Dist). Strong specificity was observed, only 2 of 54 motoneurons showing excitatory postsynaptic potentials (EPSPs) from both Lat and Dist. No EO motoneurons showed an EPSP from Dist, and no IIm motoneurons showed one from Lat. Expiratory Dist motoneurons fell into two groups. Those with Dist EPSPs and none from Lat (*group A*) were assumed to innervate distal internal intercostal muscle. Those with Lat EPSPs (*group B*) were assumed to innervate abdominal muscle (transversus abdominis or rectus abdominis). Inspiratory Dist motoneurons (assumed to innervate interchondral muscle) showed Dist EPSPs. Stimulation of dorsal ramus nerves gave EPSPs in 12 instances, 9 being in *group B* Dist motoneurons. The complete absence of heteronymous monosynaptic Group I reflex excitation between muscles that are synergistically activated in expiration leads us to conclude that such connections from muscle spindle afferents of the thoracic nerves have little role in controlling expiratory movements but, where present, support other motor acts.

the monosynaptic connection between the Group I afferents of muscle spindles and spinal motoneurons is one of the best-known synapses in the CNS and is of fundamental historical importance in the understanding of synaptic mechanisms. In recent years the high precision of this connection, in terms of the motoneuron species contacted by spindle afferents from a given muscle, has been of particular value in establishing the molecular mechanisms of the specificity of synaptic connections and their ontogeny (Arber 2012). However, for many years this specificity also has been of considerable importance in trying to understand the functional role of these reflex connections. In particular, the patterns of heteronymous connections (those from the afferents of one muscle to motoneurons of another muscle) have been investigated. These studies have included muscles of the cat and monkey hindlimb (Eccles et al. 1957; Eccles and Lundberg 1958; Hongo et al. 1984) and forelimb (Clough et al. 1968; Eccles et al. 1957; Fritz et al. 1989) and the cat neck (Anderson 1977; Rapoport 1979), as well as of the human upper (Marchand-Pauvert et al. 2000) and lower (Meunier et al. 1993) limbs. For the back muscles, heteronymous excitatory postsynaptic potentials (EPSPs) have been noted but not analyzed in terms of synergies (Jankowska and Odutola 1980). The synergies revealed by the heteronymous monosynaptic afferent input have frequently been related to patterns of muscle coactivations in centrally generated movements, most notably during locomotion (see, e.g., Engberg and Lundberg 1969). However, the certainty with which the patterns of reflex connections can be directly related to particular patterns of activation varies considerably.

One of the motor acts for which the functional role of the muscle spindle and its central connections can be considered equivocal is that of respiration. On one hand, the intercostal muscles are historically significant in the development of concepts for one particular aspect of motor control in involving spindles, alpha-gamma coactivation. This was described for respiration in simultaneous studies by Sears (1963) and by Critchlow and von Euler (1963), thus showing the importance of signals from the muscle spindles of these muscles in this motor act. On the other hand, for respiratory activity as a whole the roles of muscle spindles are more obscure. The two most obligatory inspiratory muscles, the diaphragm and the parasternal, intercostal muscles (interchondral muscle), have very few spindles (Duron et al. 1978). Moreover, for the intercostal muscles, where the alpha-gamma coactivation was first demonstrated, the monosynaptic connection to motoneurons does not always show the usual agonist properties. First, although the intercostal muscles are regarded as “spindle rich” (Duron et al. 1978), they are small, and thus the total tonic depolarization available in external intercostal motoneurons from this connection is also quite small, ∼1.2 mV in the calculations of Kirkwood et al. (1982). Second, according to Sears (1964e), muscle spindle afferents in the internal intercostal nerve, which come primarily from expiratory muscles, make monosynaptic connections with 70% of external intercostal motoneurons of the same segment, which are generally inspiratory in function. That is, muscles that are antagonists in respiratory terms appear to be synergists with respect to this monosynaptic afferent excitation.

Here we have extended the analyses of the heteronymous Group I input to another pair of muscles active in respiration. These are the internal intercostal and the external abdominal oblique, which are well recognized as being close synergists during expiration. We have tested the hypothesis that this close synergy in expiration is accompanied by a synergy in excitation from muscle spindle afferents by recording intracellularly from the motoneurons of both muscles, as well as from some others, all in the same segment of the spinal cord. We looked for heteronymous monosynaptic EPSPs from the afferents of each muscle in various motoneurons and found connections that were remarkably specific but with a complete absence of monosynaptic excitation between these two particular muscles. This therefore leads us to reject the hypothesis and raises further doubts as to the role of the monosynaptic reflex in respiratory movements.

## METHODS

### 

#### The preparation.

The experimental work was carried out at the UCL Institute of Neurology. The experiments were a subset of those already reported by Saywell et al. (2007) and were conducted according to UK legislation [Animals (Scientific Procedures) Act 1986] under Project and Personal Licences issued by the UK Home Office. The data come from nine cats of either sex, weighing 2.5–4.3 kg, anesthetized with pentobarbital sodium (initial dose 37.5 mg/kg ip and then iv as required). Neuromuscular blockade was achieved by the use of gallamine triethiodide (subsequent to surgery iv, repeated doses of 24 mg as required), and the animals were artificially ventilated via a tracheal cannula with oxygen-enriched air, to bring the end-tidal CO_2_ fraction initially to ∼4%. A low stroke volume and a high pump rate (53 min^−1^) were employed so that events related to the central respiratory drive could be distinguished from those due to movement-related afferent input. The vagus nerves were intact. CO_2_ was then added to the gas mixture to raise the end-tidal level sufficient to give a brisk respiratory discharge in the midthoracic intercostal nerves (typically 6–7%). During neuromuscular blockade, anesthesia was assessed by continuous observations of the patterns of the respiratory discharges and blood pressure together with responses, if any, of both of these to a noxious pinch of the forepaw. Only minimal, transient responses were allowed before supplements (5 mg/kg) of pentobarbital were administered. The animal was supported by vertebral clamps, a clamp on the iliac crest, and a plate screwed to the skull. Rectal temperature was maintained between 37°C and 38°C by a thermostatically controlled heating blanket. Mean blood pressures, measured via a femoral arterial catheter, were >80 mmHg throughout, maintained in a few animals by occasional intravenous infusions of 5% dextran in saline.

**Fig. 1.**
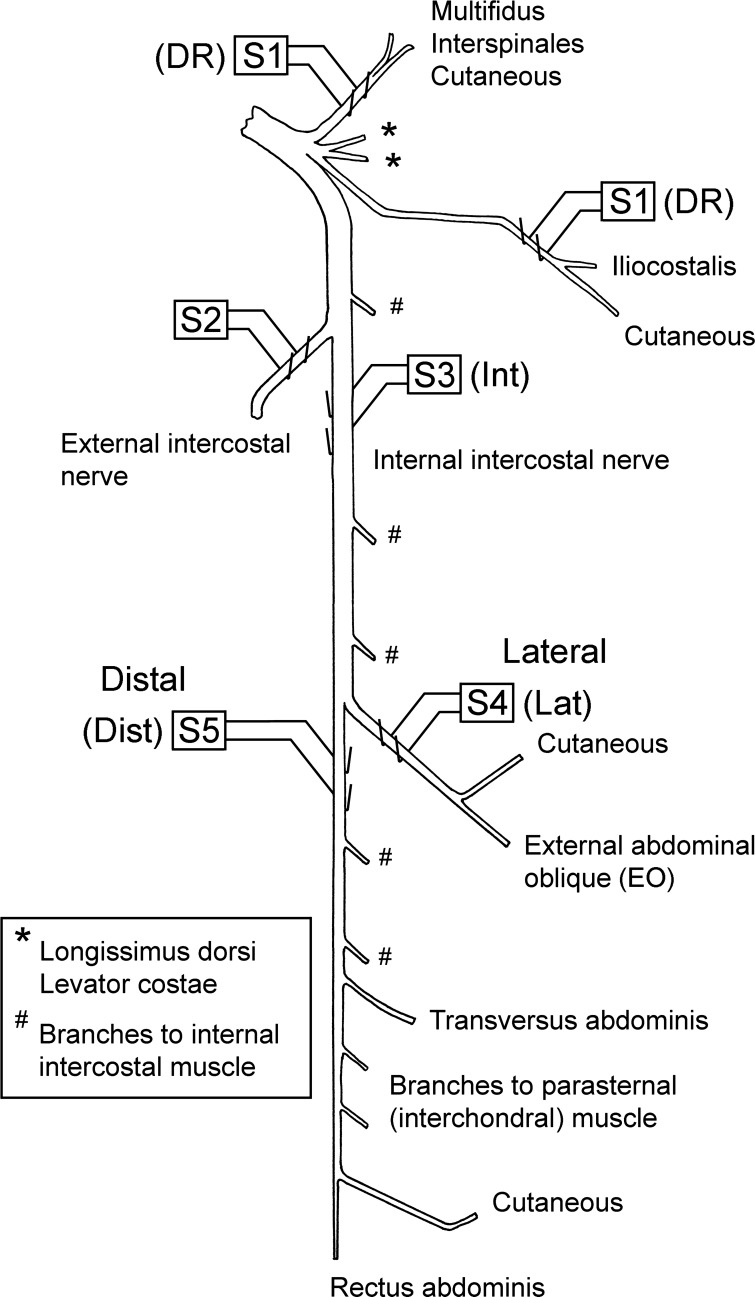
Nerve stimulation arrangement. The diagram illustrates the branching pattern for most of the nerve branches for the T_8_ segment, together with the positions of the stimulating electrode pairs, *S1–S5*. *Electrodes S3* were under the nerve, in continuity. The others were on cut nerve ends at the indicated positions. The 2 dorsal ramus (DR) nerve branches on *electrodes S1* are shown separately to indicate the approximate anatomical arrangement. In fact, the 2 branches were brought together onto a single pair of electrodes. *Electrodes S1–S3* were used for antidromic activation during tracking with the intracellular microelectrode. Motoneurons reported here were all antidromically identified from *S3* but then subsequently from *S4* [external abdominal oblique (EO)] or *S5* [distal remainder (Dist)] or from neither [internal intercostal muscle proximal to branch point (IIm), see text]. Motoneurons were tested for the presence of excitatory postsynaptic potentials (EPSPs) from stimulation of *S4* and *S5* [the main comparison, lateral branch (Lat) vs. Dist] but also from *S1* (DR) and some from *S3* [internal intercostal nerve (Int)]. The number of nerve branches shown to internal intercostal muscle (“filaments” in Sears 1964b) is arbitrary. They have not been systematically counted, but between *S3* and the lateral branch of the internal intercostal nerve there were probably 2–4, as there were probably distal to S5. Figure modified from Fig. 1 in Saywell et al. (2007), with permission. For references, see Meehan et al. (2004).

The following nerves were prepared for stimulation via platinum wire electrodes on the left side of T_8_ (numbers correspond to the stimulation sites shown in Fig. 1): *1*) a bundle of dorsal ramus (DR) nerves (Kirkwood et al. 1988); *2*) the external intercostal nerve; *3*) the most proximal point on the internal intercostal nerve (Int; in continuity but arranged to be lifted away from the volume conductor separately from the external intercostal nerve, so as to avoid stimulus spread); *4*) the lateral branch of the internal intercostal nerve (Lat); *5*) the distal remainder of the internal intercostal nerve (Dist). These nerves were used for antidromic identification of motoneurons, but here we report only those motoneurons identified from the internal intercostal nerve. Stimuli to Lat identified external abdominal oblique (EO) motoneurons (Sears 1964a), while motoneurons excited from the proximal electrodes on the internal intercostal nerve (Int) but not from either of the more distal branches (see Fig. 1) were identified as innervating the proximal part of internal intercostal or intracostalis muscles (Sears 1964a) (IIm motoneurons). Those identified from the distal remainder (Dist motoneurons) innervated the distal part of internal intercostal, transversus abdominis, rectus abdominis, or parasternal intercostal (interchondral) muscles (for references see Meehan et al. 2004). The left external intercostal nerve of T_5_ or T_6_ was prepared for recording efferent discharges, which were used to define the timing of central inspiration.

A thoracic laminectomy was made, the dura opened, small patches of pia removed from the dorsal columns of T_8_, and a shaped pressure plate lightly applied to the cord dorsum to aid mechanical stability. The laminectomy and nerves were submerged in a single paraffin oil pool constructed from skin flaps. In addition, stimulating electrodes were inserted into the left spinal cord, usually at T_10_, and an occipital craniotomy was made, both of these being required for the recording of expiratory bulbospinal neurons for the measurements of Saywell et al. (2007) but not relevant here. At the end of the experiment the animals were killed with an overdose of anesthetic.

#### Recordings.

Intracellular recordings from antidromically identified motoneurons in T_8_ with a membrane potential more negative than −40 mV were made via K^+^ acetate electrodes, stored on magnetic tape, and subsequently acquired for computer analysis (1401 A–D interface and Spike2 software, CED, Cambridge, UK). Both a low-gain d.c. version and a high-gain, high-pass filtered version (time constant 50 ms) of the motoneuron membrane potential were included. The low-gain record was used for estimating the central respiratory drive potential (CRDP; Sears 1964d) and the high-gain record, usually, for measuring synaptic potentials. Afferent nerve volleys were monitored from the cord dorsum via platinum wire electrodes mounted in the pressure plate. Both the external nerve recording and the cord dorsum recording were band-pass filtered (300 Hz–3 kHz) to remove contamination from the ECG or, for the nerves, possible movement artifacts.

#### Procedure.

The original purpose of the experiments here was the spike-triggered averaging investigation reported by Saywell et al. (2007). However, the necessary motoneuron identification routine allowed us to also record any EPSPs evoked in each motoneuron from stimulation of the two separate nerve branches, the Lat and Dist nerves. Thus the recordings presented here mostly come from the first 30–60 s of each recording (stimulation usually at a rate of 8 or 10 s^−1^), but a minute or two of the subsequent recordings with no stimulation are also considered, to help in the estimation of the CRDPs. Search stimuli were delivered to each of *nerves 1–3* above. Once a motoneuron showing an antidromic spike to the internal intercostal nerve was penetrated, these three stimuli were switched off and the motoneuron was tested in turn for antidromic activation from the Lat and Dist branches. The stimuli for these were set to 5–10 × nerve threshold, but in case of doubt (nerve thresholds can change with time) the stimulus strength was increased, so that antidromic identifications were not missed. In motoneurons without an antidromic spike to either of these stimuli (IIm motoneurons), the recordings thus necessarily included data showing the presence or absence of a monosynaptic Group I EPSP from both nerves, with stimuli at a supramaximal strength (Sears 1964c). For motoneurons identified from either nerve, the motoneuron axonal threshold was measured, so the homonymous monosynaptic EPSP at a stimulus strength just below this threshold was recorded in these instances. Note that the Dist nerve is not strictly homonymous for Dist motoneurons because this nerve innervates several muscles, so we refer to this as “homonymous.” For the IIm motoneurons, the axonal threshold from the proximal electrode on the whole internal intercostal nerve was similarly measured, so the monosynaptic EPSP from stimulation of this nerve just below threshold was similarly recorded.

#### Analysis.

**Fig. 2.**
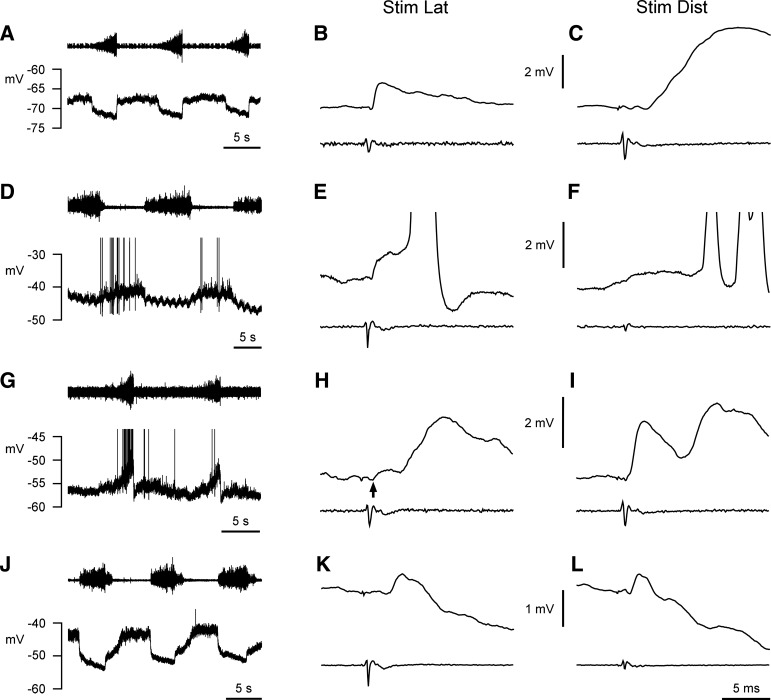
Examples of central respiratory drive potentials (CRDPs) and EPSPs. *A–C*: EO motoneuron. *D–F*: Dist motoneuron, expiratory, *group B* (see text). *G–I*: Dist motoneuron, inspiratory. *J–L*: IIm motoneuron. *A*, *D*, *G*, and *J*: CRDP for each motoneuron (T_5_ external intercostal nerve recording, *top*). *B*, *E*, *H*, and *K*: averaged responses to stimulation of Lat nerve (*bottom* trace, cord dorsum volley, upward deflection positive), stimulus strength 5–10 × nerve threshold (5T–10T), except *B*, where the volley amplitude was 51% of that at 5T–10T. *C*, *F*, *I*, and *L*: averaged responses to simulation of Dist nerve (*bottom* trace, cord dorsum volley), stimulus strength: *C* and *L*, 5T–10T; *F* and *I*, volley amplitude 72% and 100%, respectively, of that at 5T–10T. Note: monosynaptic EPSPs in *B*, *E*, *H* (amplitude 0.4 mV, arrow), *I*, and *L*; only a polysynaptic EPSP in *C*, *F*, and *K*; an additional polysynaptic EPSP in *E*, *H*, and *I*. Spikes fired by the polysynaptic components are included in the averages of *E* and *F*, which were made from the low-gain, d.c. recording. Other averages were made from the high-gain, high-pass filtered version (no spikes included). The baseline in *F* had a generally rising time course because the responses were superimposed on afterhyperpolarizations of preceding spikes. The baseline also contains (relatively low frequency) noise components. The judgment that there was no monosynaptic EPSP here relied on the absence of a fast-rising component of amplitude ≥ 0.2 mV (rise time around 1 ms) at the monosynaptic latency and superimposed on this noisy, rising baseline. Numbers of sweeps: *B*, 5; *C*, 23; *E*, 6; *F*, 7; *H*, 9; *I*, 16; *K*, 38; *L*, 52. Spikes in *D* and *G* are truncated. Voltage calibrations are common for *B* and *C*, for *E* and *F*, for *H* and *I*, and for *K* and *L*. Time calibration in *L* applies to all of the averaged responses.

The principal measurements made were the presence or absence of a monosynaptic EPSP from stimulation of each of the two nerves and, for IIm motoneurons, from stimulation of Int below axonal threshold. In addition, for all but one of the motoneurons we also had available a few seconds of recording with stimulation of the DR, used in addition to the external and internal intercostal nerves, as search stimuli during tracking for the motoneurons. The DR nerve was the first nerve stimulated in the sequence for each stimulus presentation, so there was a period of 3 ms following each DR stimulus in which the presence or absence of any monosynaptic EPSP from this nerve could be observed, uncontaminated by the responses from the other two nerves. The monosynaptic EPSPs were defined as those with central delays < 1 ms, measured from the first positive peak of the cord dorsum afferent volley. We cannot quote completely accurate values for the latencies of all of the EPSPs because the foot of the EPSPs in many instances was obscured by the antidromic field potential from nearby motoneurons (e.g., Fig. 2, *B* and *L*), but for those where this field potential was not present the latencies were between 0.40 and 0.79 ms (mean 0.61 ± 0.10 ms, *n* = 31). The amplitudes of the EPSPs were also measured, but it should be noted that some approximations were involved in this. First, because the stimulus strength was not graded to judge the maximal level, other synaptic contributions may have affected the amplitude, such as the disynaptic inhibitory postsynaptic potential (IPSP) reported to occur for stimulus strengths of ∼1.7 × nerve threshold (Sears 1964c). However, in our experience this component is small. Oligosynaptic EPSPs were also often present, readily distinguished by their central delays (1.8–10.6 ms). Occasionally, earlier low-amplitude excitatory components (central delays around 1.2–1.8 ms), representing probable disynaptic connections or connections from spindle secondary afferents (Kirkwood and Sears 1974), were seen. These components were not included in the measurements so long as a clear inflexion was present to separate them from the Group I monosynaptic component. Second, in thoracic motoneurons the monosynaptic EPSP itself is frequently modulated by respiration, being shunted by the postsynaptic inhibition that occurs in the inactive phase (Kirkwood and Sears 1973, 1982). Where possible, measurements were therefore made during the active phase. Finally, the responses to a number of stimulus presentations were averaged, but since the stimulation routine was usually performed quite quickly, sometimes only a few stimulus presentations were available for measurement. If spontaneous or orthodromically evoked spikes were present, especially during the active phase of respiration (most often expiration), individual responses with spikes present (detected by inspection) were filtered out before averaging. The end result was that the number of responses averaged varied considerably, ranging from 2 to 374 (median 18, *n* = 182). Because the motoneurons involved were active, there was frequently a high level of synaptic noise, so the measurements were accurate, at best, to the nearest 0.1 mV. This is especially true for statements about any absence of an EPSP (see results). All averages counted as showing no EPSP involved at least seven responses. In a few instances, where the presence of an EPSP was in doubt because of noise, the EPSP was only accepted if repeatable at the same, appropriate latency in two or three separate epochs.

Mean values are reported as ±SD.

## RESULTS

Recordings were made from 57 motoneurons, all initially identified from the proximal internal intercostal nerve. Stimulation of the two branches of this nerve then identified 13 of these as EO motoneurons, 32 as Dist motoneurons, and the other 12, by exclusion, as IIm motoneurons. Properties of most of these motoneurons have been previously described by Saywell et al. (2007), but we have been able to include some recordings that were too brief for their data to be included in that report and some of the motoneurons in that report could not be used for the analyses here. Furthermore, the values of CRDP and membrane potential were independently measured here, so that the values corresponded in time to the EPSP measurements. Thus the mean values quoted here are not exactly the same as in that study. Membrane potentials were estimated at the start of expiration, as in Saywell et al. (2007), and varied from −40 to −76 mV (mean −53 ± 9.1 mV). The majority of the CRDPs were expiratory (showing a depolarizing ramp during expiration and a presumed inhibitory wave during inspiration), a few were inspiratory (showing a depolarizing ramp during inspiration), one was identified as expiratory decrementing (E_dec_) as in Saywell et al. (2007), and one showed no CRDP. The CRDPs and their distributions among the three categories of antidromic identification are shown in Table 1, and examples are included in Fig. 2. Note that all the inspiratory motoneurons were in the Dist category.

**Table 1.** Properties of CRDPs in 5 groups of motoneurons

	EO (Exp)	IIm (Exp)	Dist (Exp)	Dist (Insp)	Dist (other)
No. of motoneurons	13	12	22	8	2
CRDP amplitude, mV					
Range	1–13.5	1.5–14	1–10.5	1–10	6 (E_dec_), 0
Mean	3.84	5.67	4.41	3.81	
SD	1.42	2.28	1.80	3.47	

All motoneurons were activated antidromically from an electrode on the proximal internal intercostal nerve. Groups were then defined by antidromic excitation either from the lateral branch of the internal intercostal nerve (EO) or from the distal remainder (Dist). Those activated from neither were deduced to innervate the proximal region of the internal intercostal muscle (IIm). Exp, expiratory central respiratory drive potential (CRDP); Insp, inspiratory CRDP. All the CRDPs in the EO and IIm groups were expiratory. In addition, there was 1 Dist motoneuron with a decrementing expiratory (E_dec_) CRDP (amplitude, 6 mV) and 1 with no CRDP.

Representative examples of the EPSPs evoked from the different nerves for motoneurons in each of the main antidromic identification categories are also included in Fig. 2, and a summary of the data from all the motoneurons is included in Table 2. Note that five categories of motoneuron are shown in Tables 1 and 2. We have separated the inspiratory and expiratory groups within the Dist category, because one of the four muscles known to be innervated by the Dist nerve (and only one, the interchondral muscle) has been reported to be inspiratory in function, so the presence of an inspiratory CRDP probably corresponds to an anatomical identity (see discussion). The two Dist motoneurons that did not fit these categories (1 E_dec_, 1 with no CRDP) are listed as “Dist (other).” Note also that we have only considered EPSPs of amplitude ≥ 0.2 mV. Although one or two averaged records did have the appearance of EPSPs with amplitudes below this, we were uncertain of their reliability, first because of the presence of spontaneous synaptic noise but second because the timing of their rising phases overlapped with the end of the antidromic field potential recorded extracellularly from neighboring motoneurons and it was not always easy to separate these two. Extracellular controls for the EPSPs were not recorded. In any case, such controls would have been likely to be of limited value: in our experience the antidromic field potentials, in particular, often vary considerably over very short distances in the thoracic ventral horn.

**Table 2.** Frequencies of occurrence and amplitudes of EPSPs for the 5 groups of motoneurons

	EO	IIm	Dist (Exp)	Dist (Insp)	Dist (other)
Stimulate Lat nerve					
EPSP occurrence	13/13	0/11	11/22	1/7	1/1
EPSP amplitude, mV					
Mean	0.98		1.03	0.4	0.8
SD	0.65		0.68		
Stimulate Dist nerve					
EPSP occurrence	0/13	11/11	12/22	8/8	2/2
EPSP amplitude, mV					
Mean		0.85	1.16	1.41	1.4, 4.0
SD		0.67	0.60	0.55	
Stimulate DR nerve					
EPSP occurrence	1/13	2/10	9/22	0/8	0/2
EPSP amplitude, mV					
Mean	0.6	0.2, 0.3	0.39		
SD			0.46		

One Dist (Insp) motoneuron and the Dist motoneuron with an E_dec_ CRDP were not tested with Lat stimulation. Both showed excitatory postsynaptic potentials (EPSPs) from the Dist nerve, amplitudes 1.4 and 4.0 mV, respectively.

Lat, lateral branch; DR, dorsal ramus.

The main result in Table 2 is striking. All of the EO motoneurons received an EPSP from Lat (homonymous) but none from Dist. All of the IIm motoneurons received an EPSP from Dist but none from Lat. In addition, all of the IIm motoneurons received an EPSP from Int, equivalent to those reported by Sears (1964c, 1964e). All of the inspiratory Dist motoneurons showed an EPSP from the “homonymous” Dist, and one of them also received a small EPSP (amplitude 0.4 mV) from Lat. This particular EPSP may be seen as the low-amplitude deflection (arrow) occurring before the larger oligosynaptic component (central delay 3.5 ms) in Fig. 2*H*. The situation for the expiratory Dist motoneurons was mixed. Only 12 of 22 (55%) of these showed an “homonymous” EPSP, but 11 of 22 (50%) showed an EPSP from Lat. One caveat must be mentioned: one motoneuron, initially identified as IIm, is excluded from Table 2. Unlike all the other IIm motoneurons it not only showed an EPSP from Lat (1.3 mV in amplitude) but also showed no EPSP from Dist. This motoneuron was recorded near the end of a long experiment, at a time when the thresholds for activation of both of the nerve branches had increased severalfold, and one Dist motoneuron had shown intermittent failures of antidromic activation. We therefore think that this motoneuron most likely had its axon in one of the nerve branches but its conduction was blocked peripherally. It was therefore removed from consideration.

**Fig. 3.**
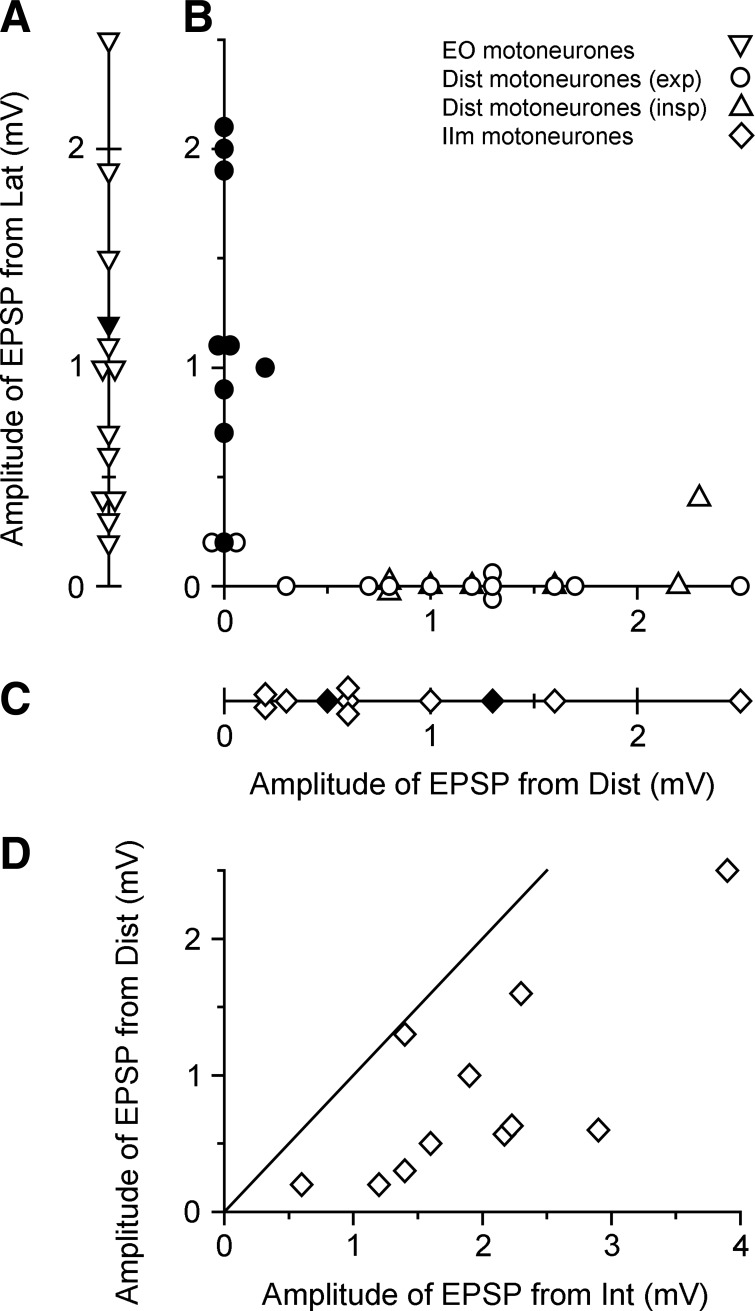
Relationships between monosynaptic EPSP amplitudes. *A*: distribution of amplitudes for the homonymous EPSPs in EO motoneurons (none of these motoneurons showed a monosynaptic EPSP from Dist nerve). *B*: amplitudes of monosynaptic EPSPs from Lat nerve plotted against the “homonymous” EPSPs in Dist motoneurons (circles, expiratory motoneurons; triangles, inspiratory motoneurons). *C*: distribution of amplitudes of monosynaptic EPSPs in IIm motoneurons from stimulation of Dist nerve (none of these motoneurons showed a monosynaptic EPSP from Lat nerve). Filled symbols in *A–C*, motoneurons with monosynaptic EPSPs from DR stimulation. *D*: amplitudes of monosynaptic EPSPs in IIm motoneurons from Dist nerve plotted against those from the proximal internal intercostal nerve (Int). Line is the line of identity.

Given the selective connectivity seen for the IIm and EO motoneurons, it is not surprising that the connectivity of the expiratory Dist motoneurons was mixed, since this category includes some motoneurons innervating intercostal and some innervating abdominal motoneurons. Moreover, the variation in connectivity was systematic. First note that the occurrence of a high percentage showing no “homonymous” EPSP (10/22, 45%) was mostly not the result of the motoneurons having low axonal thresholds: 8 of these 10 had thresholds at stimulus values where the amplitude of the afferent volley was ≥45% of its maximal value, a level close to the value where the monosynaptic EPSPs in internal intercostal motoneurons were maximal in Sears (1964c). Moreover, all of the motoneurons that did not show an “homonymous” EPSP did show an EPSP from Lat. This reciprocity is illustrated in Fig. 3*B*, where the amplitude of the EPSP from Lat is plotted against the amplitude of the “homonymous” EPSP for Dist motoneurons, The motoneurons fall into two clear clusters, the first showing a reasonable-sized EPSP from the “homonymous” nerve but none from Lat (*group A*) or vice versa (*group B*, this group also including 1 motoneuron with a 0.2-mV EPSP from Dist). The inspiratory Dist motoneurons have been included in the same plot and can be seen to correspond closely to *group A*. The end result is a very high degree of specificity. Only 2 of 54 motoneurons tested with both nerves showed EPSPs from both.

The amplitudes of the CRDPs were larger for *group A* than for *group B* (medians 5.5 and 3.5 mV, respectively), but not significantly so (Mann-Whitney, *P* > 0.05).

The amplitudes of the EPSPs were generally similar across the groups (Table 1), with the possible exception of the Dist EPSPs in IIm motoneurons (mean 0.85 ± 0.67 mV, *n* = 11). Full comparisons of the amplitude distributions are shown in Fig. 3, including the amplitudes of the homonymous EPSPs in EO motoneurons (Fig. 3*A*), for comparison with the Lat EPSPs in the Dist motoneurons, and the amplitudes of the Dist EPSPs in the IIm motoneurons (Fig. 3*C*), for comparison with the “homonymous” Dist EPSPs. The two populations of Lat EPSPs are very similar (the same median amplitudes, 1.0 mV), but the Dist EPSPs in the IIm motoneurons were generally smaller than those in the *group A* Dist motoneurons (as above, medians 0.6 vs. 1.3 mV), although the difference was not significant (Mann-Whitney, *P* > 0.05). In Fig. 3*D* we have plotted the amplitudes of Dist EPSPs against the EPSPs from stimulating the proximal whole internal intercostal nerve (Int) in the same motoneurons. All of the points lie below the line of equality, the mean ratio being 0.42 ± 0.24.

Following stimulation of DR, a small monosynaptic response was seen in 12 of 55 motoneurons (Table 2). Interestingly, all but three of these motoneurons were in the *group B* Dist category (Fig. 3). None was in *group A*, and none was in the inspiratory Dist population.

The oligosynaptic EPSPs clearly present in some of the examples in Fig. 2 have not been systematically studied, needing, as they do, much more careful consideration with regard to the stimulus parameters (range, threshold, etc.) than was possible here. However, one consistent observation might be noted. Although stimulation of the Dist nerve gave no monosynaptic response in the EO motoneurons, an oligosynaptic excitation was present in every one of these cells (e.g., Fig. 2*B*), and at much more consistent latencies than for the other motoneuron groups (central delays 2.3–3.5 ms, mean 2.57 ± 0.34 ms). Similar EPSPs were noted in EO motoneurons in L1 by Nakata et al. (1994) from stimulation of cutaneous nerve branches in the same segment.

## DISCUSSION

The principal new finding here is that EO and IIm motoneurons show a complete absence of synergy for the monosynaptic Group I afferent input. This is despite their strong synergy for expiratory excitation, as shown both by the CRDPs recorded here (also see Saywell et al. 2007) and by their efferent discharges (Road et al. 2013). They also receive common monosynaptic excitation from individual expiratory bulbospinal neurons in nucleus retroambiguus (Road et al. 2013; Saywell et al. 2007). Moreover, the motoneurons recorded here were all located in the same segment of the spinal cord and frequently very close to each other, sometimes on the same or adjacent electrode tracks.

The observations need some qualification. The assumption that the EPSPs here resulted from Group I inputs has been made by analogy with the similar EPSPs in limb motoneurons, as discussed by Sears (1964c). In these mixed nerves, there is not an anatomically derived equivalent of the Group I peak in the fiber diameter spectrum (Sears 1964a), nor is there physiological evidence for the thoracic nerves for separation of muscle spindles and tendon organs by conduction velocity, so we have avoided the use of the term “Group Ia.” However, there is little doubt that the EPSPs here were evoked by muscle spindle afferents equivalent to the Group Ia afferents in the limbs.

Sears (1964c) noted that the monosynaptic EPSP in internal intercostal nerve motoneurons was maximal at ∼1.9 × nerve threshold, so our use of stimuli of 5–10 × nerve threshold (for all except homonymous EPSPs) would always have been supramaximal. For the homonymous EPSPs, we used a stimulus strength just under motoneuronal axonal threshold, but according to Sears (1964c) this would usually have given a maximal EPSP or an EPSP within 10% of maximal. Nevertheless, there were a few motoneurons here with very low thresholds and where the homonymous EPSP would have been likely to be underestimated (there were 7/56 instances where the homonymous EPSP was measured with a volley amplitude ≤20% of maximal).

The reliability of the identification of the IIm motoneurons should be considered. This was done by exclusion, i.e., by the absence of an antidromic spike from the two more distal branches, and could therefore be seen as less reliable than the positive identification of the motoneurons of the other categories. However, first, the standard values of stimulus strength used (5–10 × nerve threshold) were higher than the value quoted by Sears (1964c) as maximal for the motoneuron antidromic field potential (3.75 × nerve threshold), which itself corresponds well to our own observations in many years' experience in this laboratory (e.g., Kirkwood et al. 1981). Second, for IIm motoneurons, checks were also made by increasing the stimulus strength. Third, in 17 of 20 instances, a polysynaptic EPSP was seen, these EPSPs having thresholds at or above the strength maximal for motoneuron antidromic activation. Finally, the clarity of the main result itself argues for successful identification of IIm motoneurons and also justifies our decision to exclude one motoneuron as being misidentified on account of axonal conduction failure.

### 

#### Interpretation of the observations.

The connections revealed in Table 2 and Fig. 3 show remarkable specificity, in that only 2 of 54 motoneurons showed EPSPs from both of the two nerves Lat and Dist. However, Dist innervates four different muscles (Fig. 1), so the question of which afferents and which motoneurons these connections represent, in terms of the individual muscles, is considered below. These interpretations are summarized in Table 3.

**Table 3.** Summary of suggested connections

		Motoneurons					
				Dist (Exp)			
Nerve Stimulated	Afferents from	EO	IIm	IIm distal (*group A*)	TA (*group B*)	RA (*group B*)	Dist (Insp) (parasternal)
Lat	EO	**+**	**0**	(0)	(+)	(+?)	**0**
Distal							
	IIm distal	(0)	(+)	(+)	(0)	(0?)	(+)
	TA	——————————————————————–Few afferents—————————————————					
	RA	——————————————–————————Few afferents—————————————————					
	Parasternal	——————–——————Few afferents——————————————					(+)
DR	DR	**0**	**0**	(0)	(+)	(+?)	**0**

Bold entries show observations that were unambiguous with regard to motoneuron or afferent muscle identity, **+** indicating presence, **0** indicating absence of connections. Normal font entries in parentheses indicate connections for motoneurons or afferents presumed to innervate the different muscles innervated by Dist, as discussed in the text. Similarly, the assignments of Dist (Exp) motoneurons to *group A* or *B* and the presence of “few afferents” from particular muscles are as suggested in the text. TA, transversus abdominis; RA, rectus abdominis. Question marks assigned to RA motoneurons reflect the view that only TA can be assigned to the particular functional synergy with a DR innervated muscle suggested in the text, yet nearly all of the *group B* motoneurons showed an EPSP from stimulation of DR; perhaps, therefore, there were very few RA motoneurons and connections to them would not then have been adequately tested. Minor categories of identification via the CRDP and low-percentage connections are omitted.

Two groups of motoneurons were well defined as each innervating a single muscle, EO and IIm (justified above). From these, one group of afferents (those from EO) could be selectively stimulated, and these gave the most secure result: a total absence of synergy, i.e., no EPSPs seen in IIm motoneurons from these afferents. However, synergy (or absence of it) may in general be bidirectional or unidirectional (Eccles et al. 1957; Fritz et al. 1989). Was the absence of synergy here bidirectional? The equivalent afferents from the proximal part of IIm could not be selectively activated, but we suggest that absence of synergy was indeed bidirectional, because a surrogate result was obtained, the total absence of EPSPs from stimulating Dist nerve. The justification for accepting this result as equivalent is as follows. This nerve would be expected to contain a number of muscle spindle afferents from the more distal part of the internal intercostal muscle, the number of the filaments that branch off this nerve to innervate internal intercostal muscle being similar to the number of filaments that branch off the main nerve proximal to the stimulation point (Fig. 1). This expectation was then supported by the EPSPs universally observed from stimulation of this nerve in the IIm motoneurons. The amplitudes of these EPSPs were relatively small (on average 42% of the amplitudes of the EPSPs from the proximal nerve stimulation) (Fig. 3*D*), but this is not unreasonable for what one might expect on the basis of the Dist branch containing this fraction of the homonymous afferents for the whole intercostal space of the internal intercostal muscle. One can thus reasonably surmise that stimulation of the Dist nerve should indeed test connectivity from internal intercostal muscle afferents, even though these originated from a more distal region than that defined here as IIm. Thus the absence of EPSPs from Dist in EO motoneurons represents a bidirectional absence of projections.

Next, we consider the observations on the expiratory subgroup of Dist motoneurons. Figure 3*B* shows that these can be split into two further subgroups: one received EPSPs only from Dist (*group A*) and the other largely only from Lat (*group B*). The most obvious interpretation of these observations is that the motoneurons of *group A* innervated the distal part of internal intercostal muscle whereas those of *group B* innervated abdominal muscles (either transversus abdominis or rectus abdominis). In support of this, the amplitudes of the EPSPs from Dist nerve recorded in *group A* were within the same range as those in the IIm group (although they had a nonsignificantly larger median), and therefore probably represent excitation from the same population of homonymous afferents. The question of identifying homonymous afferents for the motoneurons of *group B* presents more of a problem. All but one of these motoneurons showed no homonymous EPSP. This high proportion is very unusual. A somewhat similar situation occurred with respect to external intercostal motoneurons, for 30% of which Sears (1964e) detected no homonymous EPSP. Kirkwood and Sears (1982) suggested that this proportion could have arisen by chance, because of the relatively few afferents and motoneurons innervating this small muscle, combined with a relatively long segment length (10 mm) and wide spacing of afferent collaterals within the spinal cord. Perhaps this was also the case here, but the proportion without an EPSP here (10/11) was three times as high. A reasonable number of these motoneurons were recorded, so there is no evidence that their number in this segment is very low. We therefore suggest that the number of muscle spindle primary afferents in this part of these two abdominal muscles may be particularly low, or perhaps they enter the cord in other segments. For transversus abdominis, support for the idea that the afferents might be very few comes from the basic anatomy. Anatomy texts sometimes refer to the most rostral part of this muscle (middle thoracic segments) as being continuous with the most caudal parts of triangularis sterni, which itself is a muscle with very few muscle spindles (none found by Duron et al. 1978). One other observation is consistent with a scarcity of these afferents. This is the total absence of EPSPs from Dist in the EO motoneurons. Thus we are suggesting that the absence of EPSPs from Dist in either *group B* Dist or EO motoneurons may have two different components: one component related to the afferents from internal intercostal muscle, which we believe to be relatively common and for which the absence of EPSPs reflects an absence of synergy, and a second component representing afferents from the abdominal muscles, where the absence of EPSPs reflects a scarcity of these afferents (Table 3).

The final group to consider consists of the inspiratory motoneurons in the Dist group. A puzzle arises here. In previous publications from this laboratory (Kirkwood and Sears 1978; Saywell et al. 2007; Vaughan and Kirkwood 1997) it was assumed that internal intercostal nerve motoneurons that showed either inspiratory discharges or an inspiratory CRDP innervated interchondral muscle (also see Lipski and Martin-Body 1987), since this muscle is well known to be inspiratory in function (De Troyer et al. 2005; Taylor 1960). One feature of this muscle is that, like the diaphragm, it has few spindles (Duron et al. 1978). Yet the median “homonymous” EPSP amplitude for this group was as high as any (1.3 mV). Several explanations are possible. First, although the afferents might be few, they could make stronger or more widespread connections than the other thoracic spindle afferents. Second, it could be that spindles in supposed expiratory muscles might synapse on these motoneurons, as may be assumed for the external intercostal nerve motoneurons receiving EPSPs from internal intercostal nerve afferents (Sears 1964e). A likely muscle to provide such afferents might be the most ventral part of the internal intercostal muscle, which is contiguous with the most lateral part of the interchondral muscle and, at least at its border, must have the same mechanical action. Finally, by the same token, note that the definitions of areas of the intercostal/interchondral muscles that are inspiratory or expiratory, either in terms of mechanical action or in terms of activity, have only been defined in quite broad terms (De Troyer et al. 2005), so a sharp borderline should not be expected, either in using the CRDP to define the region innervated by a motoneuron or, in this respect, in the precision in the specificity of the afferent projections.

#### Functional relevance.

The earliest assignments of function to heteronymous monosynaptic connections came from Lloyd (1946), who assessed the connections for the simplest, close anatomical synergists at one joint, collectively described (with their antagonists at the same joint) as the myotatic unit. With the advent of the greater sensitivity of intracellular recording, heteronymous connections with less obvious anatomical synergies were revealed, such as that from quadriceps to soleus (Eccles et al. 1957; Eccles and Lundberg 1957; Hongo et al. 1984; Meunier et al. 1993). An important process of then assigning functions to these new connections consisted of studying natural movements, notably posture and locomotion, and observing phases of coactivation of the muscle pairs concerned. The connections could then be assigned as supporting that observed synergy. This is a correlative process, and therefore inevitably uncertain. For the forelimb, where even more disparate connections have appeared, recourse has then been made to the greater variety of assumed synergies involved in manipulative behavior (Fritz et al. 1989; Marchand-Pauvert et al. 2000) but without, in many cases, observations of the actual coactivations. However, one particular hypothesis from Lundberg (1969) should be mentioned, in which he assigned the heteronymous Group I monosynaptic connections to being part of a mechanism whereby an assumed basic flexor-extensor pattern was converted into the more complex pattern of flexions and extensions during a natural step of the hindlimb (also see Engberg and Lundberg 1969). It is not clear what the equivalent mechanism for respiration might be, but one possibility is the conversion of a basic inspiratory or expiratory output from the medulla into a two-dimensional pattern across the surface of the thorax. This appears to occur at the spinal cord level and is an obvious candidate for a peripheral afferent effect. However, it actually occurs centrally, independently of such an input (De Troyer et al. 2005).

In the present study, because our conclusion is a negative one, it can be made with a great deal of certainty. The synergy of activation between the internal intercostal and EO muscles in expiration is very clear, as is the absence of heteronymous EPSPs between this pair of muscles. Thus heteronymous monosynaptic connections do not support this activation synergy. We suggest that this may be true for expiration in general, and probably also for respiratory movements in general.

A similar conclusion, that the heteronymous monosynaptic connections for abdominal muscles do not support respiration, has in fact been made before, by Iscoe (2000), who used poststimulus histograms to study the monosynaptic responses in abdominal nerves. His conclusion was based on the absence of a contralateral response, in contrast, he argued, to respiration, which is represented bilaterally. We suggest that our conclusion is considerably stronger, because the expectation of bilateral monosynaptic connections for these muscles should not in any case be high, despite the claim from Beith and Harrison (2004) that the contralateral short-latency human abdominal stretch reflexes that they observed were monosynaptic in origin.

One of the connections observed here might seem to contradict this general conclusion. The EPSPs that we observed in the *group B* Dist motoneurons from stimulating Lat represent a connection between two expiratory muscles. However, assuming that these motoneurons innervated other abdominal muscles, and in view of the well-recognized postural roles of the abdominal muscles (e.g., Thorstensson et al. 1985), we suggest instead that this connection has a postural rather than respiratory significance. With regard to posture, two other points could be mentioned. First, EO and IIm muscles may face different loads. In a standing animal, EO can be regarded as an antigravity muscle, helping to support the abdominal contents, where IIm is not. Second, these two muscles can also be seen as acting in series. In the particular circumstance of a closed glottis, contraction of EO will stretch IIm, and vice versa.

The functional significance of the only other positive observation of a heteronymous connection, from DR nerve to the *group B* Dist motoneurons, is not immediately clear but may also have a postural significance. Perhaps the most obvious anatomical synergy from the muscles innervated from the DR bundle involves the most lateral of these muscles, iliocostalis. At the thoracic level, this consists of a narrow strip of muscle connecting the outer aspect of all the ribs at 2–3 cm lateral to the spine. This might a priori be expected to show a synergy with the underlying proximal intercostal muscle (external or internal), here represented by the IIm motoneurons, or with EO motoneurons, EO muscle arising from the outer aspect of the ribs just a little more laterally. Such synergies might be expressed in lateral flexion movements of the thorax. However, only 3 of 23 EPSPs were seen in these two groups together (Table 2). In contrast, the medial muscles supplied by the DR nerve bundle (the thoracic multifidus and interspinales) connect only between vertebrae and are involved in extension of the spine, trunk rotation, lateral flexion, and stabilization of the spine. An anatomical synergy between these muscles and the ventral abdominal muscles that we assume to be included in *group B* seems a priori remote. However, there is one possibility. Contraction of transversus abdominis in the pig was shown by Hodges et al. (2003) to increase the stiffness of the lumbar spine, consistent with the effect of increased abdominal pressure in humans (Hodges et al. 2005). This implies an extensor moment on the spine, thus suggesting that transversus abdominis could act as an agonist to the lumbar multifidi (also see Cook et al. 2009). If this was also the case for the lower thoracic segments, then the presence of EPSPs in *group B* Dist motoneurons could be understood, on the assumption that these motoneurons innervated transversus abdominis. Note that the other muscle possibly innervated by *group B* Dist motoneurons, rectus abdominis, is a definite antagonist of the dorsal paraspinal muscles. However, whatever the explanation, our observation of such a specific connection to the *group B* Dist motoneurons is valuable in its own right, in that it gives independent confirmation of the validity of the separation of *groups A* and *B*.

Perhaps the main result, the absence of heteronymous monosynaptic connections between IIm and EO, may itself have a postural significance. For instance, in human trunk rotation movements, IIm on the right is activated with rightward rotation (Whitelaw et al. 1992) whereas EO is activated mainly with rotation to the left (for references see Urquhart and Hodges 2005). Thus a heteronymous connection here might be a disadvantage, although it is unclear by how much, since most of the studies (at least for EO) show a degree of cocontraction between the muscles of both sides of the body during rotation.

The functional significance of another negative observation, the absence of homonymous EPSPs in the *group B* Dist motoneurons, is most intriguing, even though not unique. A similar absence was noted for motoneurons innervating cutaneous trunci in the rat (Theriault and Diamond 1988) or its homolog, cutaneous maximus in the mouse (Vrieseling and Arber 2006). However, the situation differs between the rat and the mouse, in that spindles are almost absent in this muscle of the rat (Theriault and Diamond 1988) but are reasonably plentiful in the mouse (Vrieseling and Arber 2006). It could be that this muscle is regarded as having rather specialized functions, in wrinkling the skin in response to local irritation (Petruska et al. 2014) or in mating (Gerrits et al. 2000; Theriault and Diamond 1988), and that such specialized motor acts could be hypothesized to be controlled in an open-loop fashion, without sensory feedback. Might the expiratory action of the Dist *group B* motoneurons be similarly regarded? Perhaps, but one difference should be noted: in mouse cutaneous maximus Vrieseling and Arber (2006) found an absence also of heteronymous EPSPs (as tested from stimulation of the dorsal roots), whereas in the Dist motoneurons of *group B* here heteronymous monosynaptic EPSPs were present, from at least two sources. Yet more variation is seen if consideration of the neck muscles is included. These are among the muscles most richly endowed with spindles, but the projection frequencies of these afferents to motoneurons are particularly low (Keirstead and Rose 1988), including an absence of heteronymous connections in the small sample tested by these authors (but cf. Anderson 1977; Rapoport 1979).

These are disparate observations, but yet one more is provided by the relatively large EPSPs seen in the inspiratory Dist motoneurons. On the assumption that these motoneurons innervated interchondral muscle, what functional significance should this have? First, this result emphasizes again that the strength of monosynaptic excitation cannot generally be predicted from the number of spindles in a muscle. Nevertheless, the paucity of the spindle content of this muscle has been associated with an absence of a spinal stretch reflex (De Troyer 1991). In that study, the presence of a stretch reflex in the external intercostal muscle during inspiration with tracheal occlusion was contrasted with a vagal inhibition in the interchondral muscle. It is instructive that, if our result at T_8_ was also applicable to the segments used by De Troyer (probably T_2_–T_6_), then it could be that this major difference in a functional reflex occurred for two motoneuron pools that actually received a very similar monosynaptic input. The implication then would be that the functional stretch reflex concerned did not significantly depend on monosynaptic connections but on more complex circuitry.

This last point also serves as a reminder that our main conclusion, that heteronymous monosynaptic connections have no role in supporting expiratory movements, should not be extrapolated too far. What we must not conclude is that the spindle afferents themselves have no role, since they participate in a variety of other pathways. Furthermore, the other disparate observations discussed above, which do not seem to fit easily with the previously suggested roles for heteronymous connections derived from studies of hindlimb muscles, emphasize how little we actually understand about the functions of these connections. For the functional roles of spindle connections in general, observations of natural movements after spindle inputs to motoneurons have been removed are hard to achieve acutely. Recent development of mutant mice models, either without spindle projections to the ventral horn (Arber et al. 2000) or with disruptions of these projections (Arber 2012), could contribute new insights, although even then the distinction between the functions of the homonymous versus the heteronymous connections may remain hard to achieve. Thus we will probably still need to rely on correlative approaches. In one of the earliest discussions on this matter, with regard to locomotion, Engberg and Lundberg (1969) stated, “Before closing this discourse on stepping we would like again to emphasize its entirely speculative character.” This is still the situation we are in (Windhorst 2008), but to make any advances using the correlative approach, the widest possible range of muscles and motor acts should be considered. In this respect the present observations, involving respiratory movements and a clear negative correlation, should be particularly valuable.

## GRANTS

The work was supported by the International Spinal Research Trust and the Jeanne Anderson Fund (Institute of Neurology). C. F. Meehan held a Medical Research Council (MRC) studentship.

## DISCLOSURES

No conflicts of interest, financial or otherwise, are declared by the author(s).

## AUTHOR CONTRIBUTIONS

Author contributions: T.W.F., C.F.M., and P.A.K. performed experiments; T.W.F., C.F.M., and P.A.K. interpreted results of experiments; T.W.F., C.F.M., and P.A.K. edited and revised manuscript; T.W.F., C.F.M., and P.A.K. approved final version of manuscript; P.A.K. conception and design of research; P.A.K. analyzed data; P.A.K. prepared figures; P.A.K. drafted manuscript.
